# Optimally fixed F protein on the surface of RSV-infected cells for RSV binding and neutralizing assays

**DOI:** 10.3389/fmicb.2026.1771336

**Published:** 2026-03-04

**Authors:** Hongsheng Qiang, Yujin Shi, Zemin Jiang, Yuhao Fu, Xiaobin Zhang, Li Chen, Yongpeng Sun, Yangling Wu, Zizheng Zheng, Ningshao Xia

**Affiliations:** 1 State Key Laboratory of Vaccines for Infectious Diseases, Xiang An Biomedicine Laboratory, Department of Laboratory Medicine, School of Public Health, Xiamen University, Xiamen, Fujian, China; 2 National Institute of Diagnostics and Vaccine Development in Infectious Diseases, School of Public Health, Xiamen University, Xiamen, Fujian, China; 3 Emergency Department, The First Affiliated Hospital of Xiamen University, Xiamen, Fujian, China; 4 Department of Clinical Laboratory, Xiang'an Hospital of Xiamen University, Xiamen University, Xiamen, Fujian, China

**Keywords:** cell-based ELISA, RSV, F protein, binding antibody titer, neutralizing antibody titer

## Abstract

Respiratory syncytial virus (RSV) presents a significant global public health challenge, contributing substantially to the disease burden worldwide. As numerous F protein-based vaccine candidates advance into clinical trials, robust evaluation methods are essential. Here, we developed a cell-based ELISA platform to rapidly evaluate serum antibody responses to these vaccines. By treating RSV-infected cells with 4% formaldehyde or 60% methanol, the cell-surface F proteins were stabilized in the pre-fusion (pre-F) or post-fusion (post-F) conformations, respectively. This platform efficiently determines the binding and neutralizing activities of post-immunization serum antibodies, providing an effective method for evaluating the efficacy of RSV vaccine candidates.

## 1 Introduction

Respiratory syncytial virus (RSV) is a major leading cause of acute lower respiratory infections (ALRIs) among children under 5 years of age and elderly individuals. Particularly among young infants, RSV poses a higher risk than influenza, significantly contributing to infant mortality worldwide (Gill et al., 2022; Hansen et al., 2022). To date, three RSV vaccines developed by GSK, Moderna, and Pfizer have been successfully approved (Kingwell, 2023). These vaccines, however, are primarily designed to prevent RSV infections in elderly individuals and pregnant women. While passive immunization therapies have been authorized for high-risk infants (Cetinkaya et al., 2017; Hammitt et al., 2022; Moline et al., 2025), the development of a safe and broadly applicable RSV vaccine for wider population use remains an essential objective in global public health. Therefore, there is a critical need for robust methods to rapidly assess the effectiveness of RSV vaccine candidates, especially against the diverse wild-type RSV strains.

The RSV surface fusion (F) glycoprotein, which displays more neutralizing epitopes and is highly conserved compared to the G glycoprotein (Melero and Moore, 2013), provides an effective target for vaccine and monoclonal antibody development. During the viral entry process, the F protein transitions from a metastable pre-F conformation to a stable post-F conformation (Collins and Melero, 2011). The pre-F conformation, which retains neutralization-sensitive sites absent in the post-F conformation, is the major target recognized by serum neutralizing antibodies from naturally infected individuals with RSV (Magro et al., 2012; Mclellan et al., 2013; Ngwuta et al., 2015). This understanding has driven advancements in vaccine antigen design, with the pre-F protein emerging as a crucial immunogen and a key metric for evaluating immunogenic efficacy, particularly regarding the induction of potent neutralizing antibodies (Ngwuta et al., 2015; Mukhamedova et al., 2021; Taleb et al., 2021). Although soluble pre-F proteins [e.g., DS-Cav1 (Mclellan et al., 2013), DS2 (Joyce et al., 2016) and SC-TM (Krarup et al., 2015)] have been stabilized by structure-based design. Expression and purified production of high-purity pre-F trimer of RSV clinical strains remains a challenging and time-consuming process, especially for evaluating serum binding titers post-vaccination.

The neutralization assay is a critical measure for assessing the efficacy of RSV vaccine candidates. Although the classical plaque reduction neutralization assay is considered a highly reliable method, its complexity and time-consuming nature limits its suitability for high-throughput screening. Consequently, many studies have turned to genetically engineered RSV strains (Bakkers et al., 2024; Gidwani et al., 2024; Lin et al., 2025) incorporating fluorescent reporters to simplify the quantification of neutralizing antibody titers, but these methods are limited in their applicability across diverse RSV clinical isolates.

In a previous study, we reported that the F protein expressed on the surface of RSV-infected cells could be maintained in a pre-fusion conformation using appropriate fixation conditions (Zhang et al., 2019). Based on this foundation, we developed a cell-based enzyme-linked immunosorbent assay (ELISA) utilizing the F protein on the surface of RSV-infected cells treated with optimized fixation methods. Our results demonstrate that 4% formaldehyde or 60% methanol can effectively stabilize cell-membrane F proteins in pre-F or post-F states, respectively, while preserving intact antigenic epitopes. Subsequently, we established a cell-ELISA platform to assess the binding and neutralizing activities of RSV antibodies, as well as serum antibody responses following immunization with pre-F (DS-Cav1) or post-F proteins.

## 2 Materials and methods

### 2.1 Cells and virus strains

HEp-2 cells (ATCC, CCL-23™) were cultured in Minimum Essential Medium (MEM; Thermo Fisher Scientific, Waltham, MA, USA, Cat. #42360099) supplemented with 10% inactivated fetal bovine serum (Thermo Fisher Scientific, Cat. #10099141) and 100 U/ml penicillin–streptomycin (Thermo Fisher Scientific, Cat. #15140-122). The RSV A2 strain (ATCC), recombinant virus, A2-mKate and RSV clinical isolates (7445 and 7127) were produced in HEp-2 cells as previously described (Rameix-Welti et al., 2014) and stored at −80 °C to ensure the stability. Viral titers were determined via plaque assay as previously mentioned (Zhang et al., 2019).

### 2.2 Antibodies and proteins

Expression and purification of soluble RSV-F proteins and antibodies against the F protein were performed according to previous methods (Mclellan et al., 2013). In brief, plasmids encoding the pre-F protein (DS-Cav1), post-F protein and antibodies against RSV F protein were transiently transfected into Expi293F™ cells (Thermo Fisher Scientific, Cat. #A14528). After 7 days, the cell culture supernatants were collected and centrifuged to remove cell debris. Ni Sepharose fast-flow 6 resin (GE Healthcare, USA) was used for RSV-F proteins, followed by further purification on HiLoad 16/600 Superdex 200 pg size exclusion column (GE Healthcare) according to the manufacturer's instructions. For antibodies against the F protein, the protein A agarose column (GenScript, Cat. #L00210) was used. Purified trimeric F proteins and antibodies were dialyzed against 1 × PBS at pH 7.4, and stored at −80 °C until further use.

### 2.3 Flow cytometry

At 24 h post-infection, HEp-2 cells infected with RSV A2-mKate were detached and prepared as single-cell suspensions. Cells were resuspended in staining buffer and incubated on ice with primary antibodies targeting distinct epitopes of the F protein (10 μg/ml) for 30 min. After two washes with staining buffer, samples were incubated on ice in the dark with the appropriate BV421-conjugated secondary antibodies (A85-1, R2-40 or G18-145, BD Bioscience https://www.bdbiosciences.com/zh-cn) for 30 min. Following final washes, cells were resuspended in staining buffer and immediately acquired on the BD LSRFortessa X-20. Data were analyzed using FlowJo software (version 10.10.0, BD Bioscience https://www.flowjo.com/company).

### 2.4 Immunofluorescence assay for RSV fusion protein

HEp-2 cells infected with RSV A2-mKate at a multiplicity of infection (MOI) of 1 were seeded in 96-well cell culture plates at 3 × 10^4^ cells per well and cultured for 24 h at 37 °C, 5% CO_2_. After gently washing twice with phosphate-buffered saline containing 0.05% Tween-20 (PBST), cells were fixed with 4% paraformaldehyde for 15 min, and then blocked for 2 h with 5% non-fat dry milk in PBS at room temperature. Hundred microliter of 0.1 mg/ml RSV F protein antibody was added to each well, incubated for 1 h at room temperature, followed by a 2,000-fold diluted anti-mouse Alexa Fluor 488 secondary antibody (Thermo Fisher Scientific, Cat. #A32723) for 1 h. Nuclei were stained with a 2,000-fold dilution of DAPI for 5 min. Cells were washed three times with PBST after each step.

### 2.5 Cell-based ELISA for binding activity assay

HEp-2 cells were infected with RSV A2 or A2-mKate at an MOI of 2, incubated at 37 °C, 5% CO_2_ for 14 h. Then, the cells were trypsinized and reseeded into a 96-well plate at 3.5 × 10^4^ cells per well, followed by an additional 8 h of incubation. The cells were gently washed twice with PBST and centrifuged to remove residual liquid. Subsequently, cells were fixed with various concentrations of formaldehyde or methanol solution at room temperature for 30 min. Following extensive washing, plates were blocked with 200 μl of blocking buffer per well for 2 h at 37 °C. Serially diluted antibodies or heat-inactivated serum were added and incubated for 1 h at 37 °C. After washing five times with PBST, plates were incubated for 1 h with HRP-conjugated goat anti-human/mouse IgG Fc secondary antibody (Abcam https://www.abcam.com/en-us, Cat. # ab97225/ab97265) at 1:5,000 dilution. Finally, the plates were developed by addition of the HRP substrate, TMB for 15 min at 37 °C, and the reaction was stopped with sulfuric acid before measuring absorbance at 450 nm/630 nm [OD_(450nm/630nm)_]. Binding titers were calculated as the antibody concentration or the serum dilution that resulted in a 50% reduction in absorbance by three-parameter logistic regression in GraphPad Prism (version 10.4.1, GraphPad Software https://www.graphpad.com) 10, expressed as EC_50_ titers or ED_50_ titers.

### 2.6 Cell-based ELISA for neutralizing activity assay

Heat-inactivated serum was diluted 1:10 (antibody at 10 μg per well) in cell culture medium and serially diluted in a fourfold series (75 μl final volume). An equal volume of RSV A2 or A2-mKate (1 × 10^5^ PFU/well) was added to each well and incubated at 37 °C, 5% CO_2_ for 1 h. HEp-2 cells were then trypsinized, counted, and seeded in a 96-well plate at 3 × 10^4^ cells, 100 μl per well. An equal volume of serum/antibody-virus mixture was added to the cells for incubation at 37 °C, 5% CO_2_ for 24–36 h. Cells were fixed with 4% formaldehyde at room temperature for 30 min. After extensive washing, the cell-plates were blocked for 2 h and then incubated with MPE8 (0.5 ng/μl, 100 μl per well) for another 1 h at 37 °C. HRP-conjugated secondary antibody (Abcam, Cat. # ab97225) was then added for a 1 h at 37 °C incubation, respectively. Plates were read for absorbance at 450 nm/630 nm [OD_(450nm/630nm)_]. Neutralizing titers were calculated as the antibody concentration or the serum dilution that caused a 50% reduction in absorbance by three-parameter logistic regression in GraphPad Prism 10, expressed as IC_50_ titers or ID_50_ titers.

### 2.7 Murine vaccinations

Female Balb/C mice (6–8 weeks old) were purchased from Shanghai SLAC Laboratory Animal Co., Ltd., China. All animals were housed in specific pathogen-free animal facilities on a 12 h light/dark cycle and the temperature was kept at a range of 20–24 °C. The mice (*n* = 5 per group) were intramuscularly primed with 5 μg of F protein with Alum (InvivoGen https://www.invivogen.com, Cat. # vac-alu-50) on day 0, boosted on day 14, and serum was collected on day 21 for neutralization and binding titers assessment against F protein. The animal study protocol was approved by the Laboratory Animal Management Ethics Committee of Xiamen University (approval no. XMULAC20230322).

### 2.8 Sera collection from healthy adult donors

Serum samples from healthy adult donors used for RSV neutralization assays were obtained with written informed consent, and the study protocol was approved by the Institutional Ethics Committee of the School of Public Health, Xiamen University (approval no. SPHIRB-202101).

### 2.9 Statistical analysis

Lines present in the graphs represent the mean as indicated. Binding and neutralizing titers were compared using unpaired Student's *t* test performed using the GraphPad Prism (version 10.4.1). Two-tailed *P* values of < 0.05 are considered statistically significant.

## 3 Results

### 3.1 HEp-2 cells infected with RSV exhibit F protein on cell membranes

**Figure 1:**
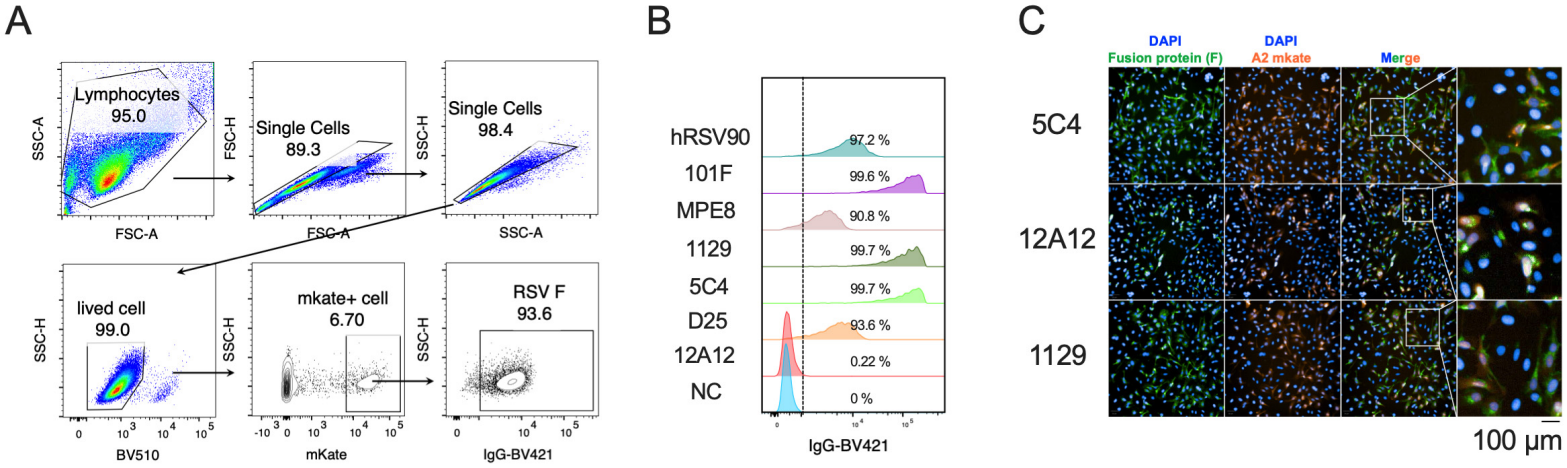
Surface expression of RSV F protein in infected HEp-2 cells. **(A, B)** Flow cytometric analysis of F protein expression on HEp-2 cells 24 h post-infection with RSV A2-mKate. Cells were stained with primary antibodies targeting distinct epitopes of the F protein, followed by fluorophore-conjugated secondary antibodies: the Flow cytometry gating strategy **(A)** and the percentage of antibody-binding cells within the mKate-positive population. NC, negative control antibody **(B)**. **(C)** Immunofluorescence analysis of RSV A2-mKate-infected HEp-2 cells. RSV A2-mKate is shown in orange. Surface F protein was detected using conformation-specific monoclonal antibodies and Alexa Fluor 488-conjugated anti-mouse IgG (green). Nuclei were counterstained with DAPI (blue). Scale bar = 100 μm.

To establish a cell-based ELISA platform for evaluating the serum binding and neutralizing titers of RSV vaccine candidates targeting the F protein, we first tested whether RSV-infected HEp-2 cells express the F protein on their membranes. Using several reported epitope-specific antibodies (D25, 5C4, hRSV90, pre-F-specific antibodies; 12A12, a post-F-specific antibody; and 1129, MPE8, 101F, antibodies binding both pre-F and post-F) against F protein, flow cytometry analysis revealed that HEp-2 cells infected with RSV A2-mKate (an RSV strain carrying a fluorescent protein gene) were efficiently bound by nearly all antibodies tested, with the notable exception of 12A12. These results confirm the successful expression of F protein on the surface of infected cells and further suggest that the F protein is predominantly presented in the pre-fusion (pre-F) conformation (Figures 1A, B).

Furthermore, we selected three F-specific monoclonal antibodies (5C4, 12A12, and 1129) to investigate the conformational states of F protein on the cell membrane after plating and fixation of infected cells with 4% paraformaldehyde in 96-well plates. Notably, we found that F protein on the membrane exhibited distinct conformational states, with both pre-F and post-F conformations being displayed (Figure 1C).

### 3.2 Different fixation methods induce favoring of specific conformational states of the F protein

**Figure 2:**
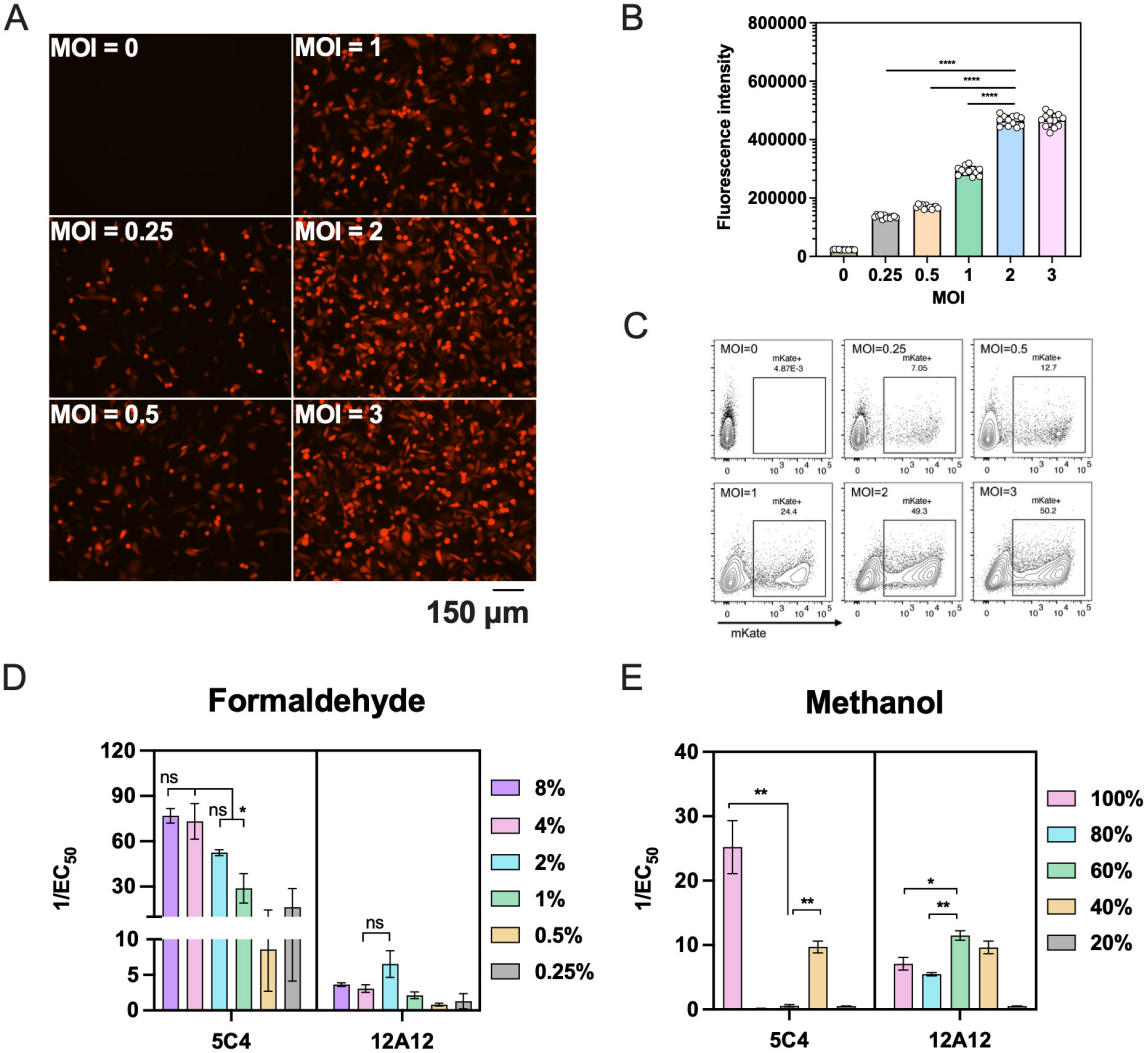
Fixation of RSV A2-mKate infected HEp-2 cells with varying concentrations of formaldehyde or methanol. **(A–C)** HEp-2 cells were infected with RSV A2-mKate at graded MOI (range: 0.25–3). Infection efficiency was quantitatively evaluated at 14 h using three methods: fluorescence microscopy imaging (A), quantification of mKate fluorescence intensity **(B)** and flow cytometric analysis for the percentage of the mKate-positive cells **(C)**. **(D, E)** HEp-2 cells infected with RSV A2-mKate at a MOI of 2 were fixed at room temperature for 30 min using different concentrations of formaldehyde **(D)** or methanol **(E)**. The conformational states of the F protein on the cell surface were assessed via immunostaining with pre-F-specific monoclonal antibody 5C4 and post-F-specific monoclonal antibody 12A12. The antibody responses to F proteins in different conformations are presented as the reciprocal binding titer [1/EC_50_ (ng/ml)]. Data are presented as mean ± SEM for three independent experiments. Statistical differences between groups were analyzed by unpaired Student's *t* test. *P < 0.05; **P < 0.01; ****P < 0.0001; ns, no significant.

To evaluate the specificity of post-vaccination sera for binding pre-F or post-F proteins, we explored various cell fixation methods to stabilize F protein on cell membranes in a predominantly single conformational state. First, we determined the optimal viral dose required to achieve near-saturating infection of cells. Using RSV A2-mKate to enable visualization of infection, we observed that an MOI of 2 produced fluorescence intensity that approached saturation relative to higher inocula (Figures 2A, B). Flow-cytometric analysis similarly indicated that infection reached a plateau at MOI = 2, although the mKate-positive fraction remained ~50% (Figure 2C).

Subsequently, we fixed infected cells with different concentrations of formaldehyde or methanol and assessed F protein conformations using 5C4 (pre-F specific) and 12A12 (post-F specific) antibodies. Low formaldehyde concentrations (0.25% and 0.5%) appeared to restrict detectable surface F largely to the pre-F conformation; however, 5C4 signals were comparatively weak under these conditions. Compared with 2% formaldehyde, fixation with 4 or 8% formaldehyde further favored the pre-F conformation, yielding stronger 5C4 reactivity while maintaining minimal 12A12 binding. Although these differences did not reach statistical significance (Figure 2D). In contrast, fixation with 60% methanol shifted surface F predominantly to the post-F conformation, abolishing 5C4 binding and producing robust 12A12 reactivity. Although 40% methanol also increased 12A12 binding, residual 5C4 binding remained detectable (Figure 2E). These results demonstrate that fixation conditions can be tuned to modulate the conformational presentation of RSV F protein on infected cells. Accordingly, we selected 4% formaldehyde and 60% methanol for subsequent experiments to preferentially present pre-F and post-F on RSV-infected cell membranes.

### 3.3 Fixed F protein retains intact antigenic epitopes

**Figure 3:**
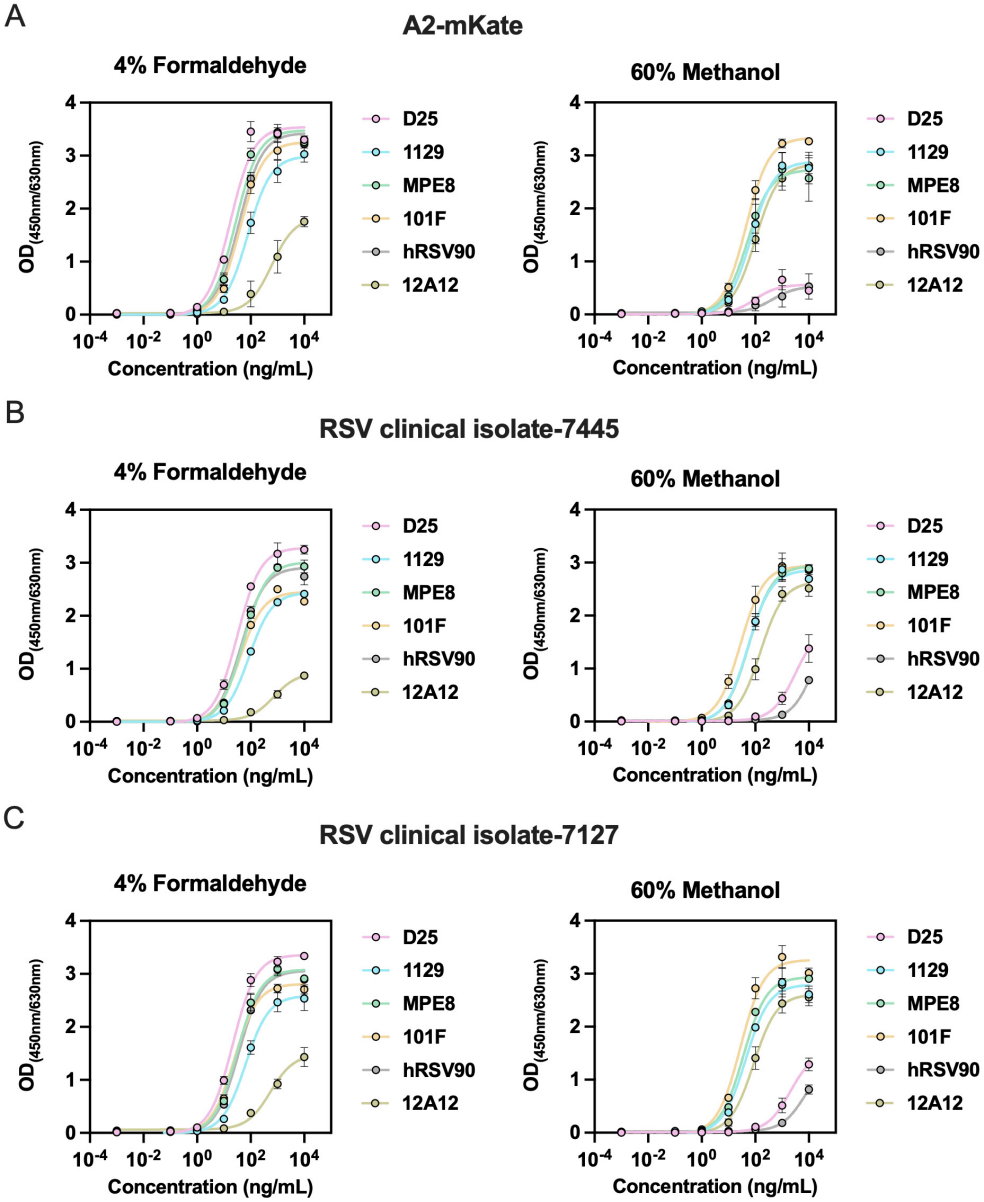
Epitope-specific antibody binding activity of RSV F protein under different fixation conditions. **(A–C)** HEp-2 cells infected with RSV A2-mkate or RSV clinical isolate (7445 and 7127) were fixed with 4% formaldehyde **(A)** or 60% methanol **(B)** to evaluate fixation-dependent epitope accessibility. Serially diluted RSV F-specific monoclonal antibodies (D25, 1129, MPE8, 101F, hRSV90, and 12A12) were applied, and binding activity was quantified using HRP-conjugated secondary antibodies with absorbance measurement at 450 nm/630 nm. Data are presented as mean ± SEM for triplicate wells.

RSV F protein contains six antigenic epitopes, including five neutralizing epitopes: site Ø and V (pre-F-specific), and sites II, III, IV (shared between pre-F and post-F). To evaluate epitope preservation post-fixation, we tested the reactivity of neutralizing epitope-specific mAbs (D25 for site Ø, 1129 for site II, MPE8 for site III, 101F for site IV, hRSV90 for site V). To assess the generalizability of the fixation conditions, HEp-2 cells were infected with multiple RSV strains, including RSV A2-mKate and the clinical isolates 7445 and 7127. Under 4% formaldehyde fixation, all five antibodies showed strong reactivity, although 1129 exhibited slightly reduced binding; the post-F–specific antibody 12A12 also retained weak binding, except in cells infected with clinical isolate 7445 (Figure 3B). These results indicates that 4% formaldehyde preferentially preserves F protein in a pre-F–enriched state while retaining a minor post-F. In contrast, with 60% methanol fixation, binding by antibodies targeting sites 0 and V was largely abolished, whereas antibodies against sites II–IV, together with 12A12, retained robust reactivity (Figures 3A–C), confirming that methanol fixation locks F protein in post-F conformation. Collectively, these data indicate that both fixation methods preserve critical neutralizing epitopes corresponding to their respective conformational states.

### 3.4 Cell-based ELISA enables rapid assessment of serum binding and neutralizing titers for RSV vaccines

**Figure 4:**
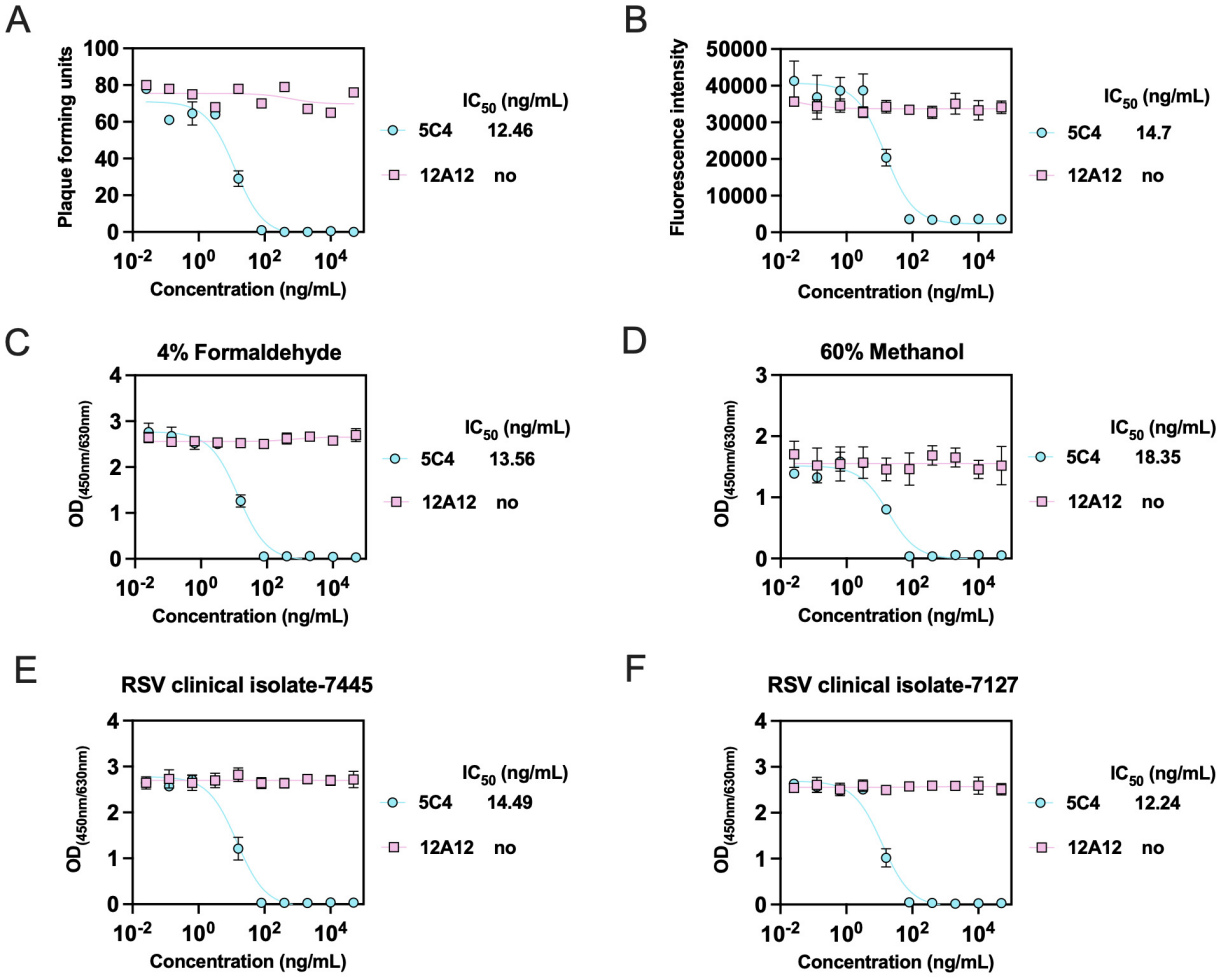
The cell-based ELISA platform for assessing neutralization against RSV. **(A–D)** HEp-2 cells were inoculated with RSV A2-mKate pre-incubated with neutralizing antibodies (5C4 and 12A12). After 3 days of incubation, cells were stained, and plaque counts were quantified to assess infection efficiency **(A)**. After 24 h of incubation, infection efficiency was quantitatively by measuring cellular fluorescence intensity **(B)**. Cells were then fixed with 4% formaldehyde **(C)** or 60% methanol **(D)** and analyzed by cell-based ELISA using the detection antibody MPE8 (0.5 ng/μl). **(E, F)** The neutralizing potency of 5C4 and 12A12 against RSV clinical isolates 7445 **(E)** and 7127 **(F)** was evaluated in HEp-2 cells fixed with 4% paraformaldehyde. Neutralizing titers were expressed as IC^50^ values (ng/ml). Data are presented as mean ± SEM for duplicate wells. no, no neutralizing activity.

Neutralizing antibody titers are a key metric for evaluating RSV vaccine efficacy. To evaluate the cell-based ELISA platform as a potential neutralization assay, fourfold serial dilutions of monoclonal antibodies 5C4 and 12A12 were preincubated with RSV A2 mKate and then applied to HEp-2 cells. Neutralization potency was determined by three independent assays. In the classical plaque-reduction neutralization assay, 5C4 showed an IC_50_ of 12.46 ng/ml against A2 mKate, while 12A12 exhibited no detectable neutralizing activity (Figure 4A). In the fluorescence-based neutralization assay (Sun et al., 2018), 5C4 effectively neutralized A2-mKate with an IC_50_ of 14.7 ng/ml, whereas 12A12 showed no activity (Figure 4B). For the cell-based ELISA method, infected cells fixed with 4% formaldehyde or 60% methanol were probed with 0.5 ng/μl MPE8 (a pre/post-F cross-reactive antibody), followed by an HRP-conjugated secondary antibody. Both fixation methods allowed determination of neutralizing titers of 5C4 and 12A12, but cells fixed with 4% formaldehyde produced substantially stronger signals and a 5C4 IC_50_ value closer to those obtained with the other two assays (4% formaldehyde: IC_50_ = 13.56 ng/ml; 60% methanol: IC_50_ = 18.35 ng/ml), indicating higher sensitivity with 4% formaldehyde fixation (Figures 4C, D). Using the 4% formaldehyde cell-based ELISA, we were also able to effectively measure neutralizing titers of 5C4 and 12A12 against RSV clinical isolates (Figures 4E, F). These results indicate that the cell-based ELISA is a viable method for assessing RSV neutralizing activity.

**Figure 5:**
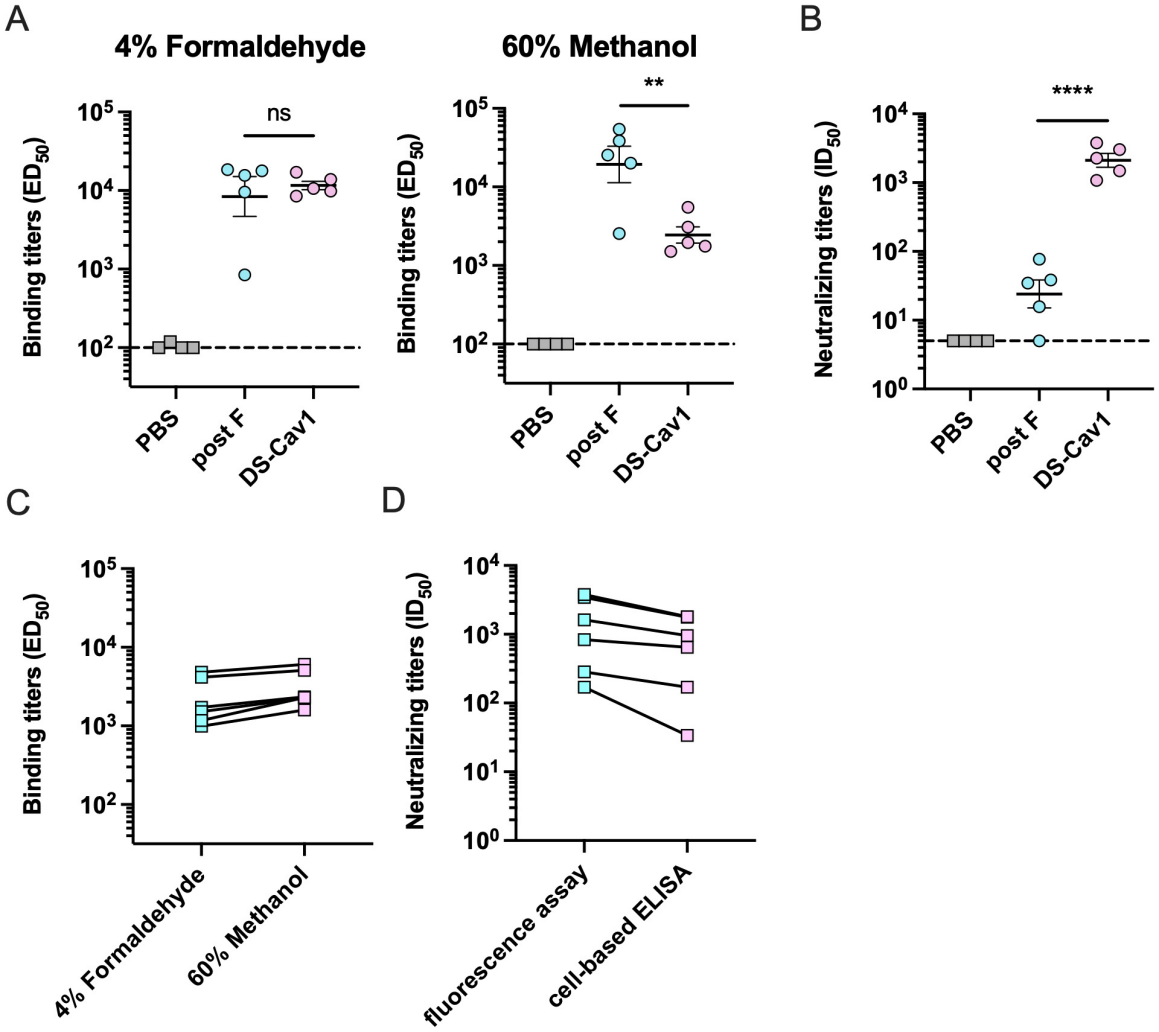
Assessment of serum binding and neutralizing titers following RSV F protein immunization. **(A, B)** Mice were immunized with a mixture of 5 μg of RSV F protein and alum (*n* = 5 per group). Serum was collected 1 week post-immunization for the assessment of binding and neutralizing antibody titers. The pre-F binding titer was measured by cell-based ELISA with RSV A2-infected cells fixed in 4% formaldehyde, while the post-F binding titer was measured using cells fixed in 60% methanol **(A)**. Additionally, the neutralizing titer was determined using a cell-based ELISA with cells fixed in 4% formaldehyde **(B)**. **(C, D)** Serum binding and neutralizing titers against RSV A2-mKate were determined for healthy adult donors. The pre-F and post-F binding titer was measured by cell-based ELISA with cells fixed in 4% formaldehyde and 60% methanol **(C)**. The neutralizing titer was determined by measuring cellular fluorescence intensity and by cell-based ELISA using 4% formaldehyde-fixed cells **(D)**. Data are presented as mean ± SEM. Statistical differences between groups were analyzed using unpaired Student's *t* test. **P < 0.01; ****P < 0.0001; ns, no significant.

To further assess the ability of the cell-based ELISA platform to reflect vaccine serum neutralization potency against RSV wild strains, Balb/c mice were immunized with 5 μg of RSV pre-F protein (DS-Cav1) or post-F protein using a two-dose regimen. Serum was collected 1 week post-boost. Treated cells infected with RSV A2 (4% formaldehyde for pre-F stabilization, 60% methanol for post-F stabilization) were used to measure binding specificity with serially diluted heat-inactivated sera. DS-Cav1-immunized sera showed significantly reduced binding to 60% methanol-fixed cells (post-F conformation) compared with 4% formaldehyde-fixed cells, indicating that the induced antibodies preferentially recognize pre-F-specific epitopes. In contrast, the post-F immunized sera showed comparable binding to both conformations (Figure 5A). Importantly, Neutralization assays against RSV A2 using the cell-based ELISA platform revealed superior activity in DS-Cav1 sera compared to post-F sera, with a difference of 100 times (Figure 5B). These data indicate that DS-Cav1 immunization induces potently neutralizing antibodies, whereas post-F immunization elicits antibodies that bind strongly but display limited neutralizing activity.

To further validate the cell-based ELISA platform for quantifying serum antibody binding titers and neutralization, we tested sera from healthy adult donors. Binding titers to HEp-2 cells fixed with either 4% formaldehyde or 60% methanol were comparable across the cohort, indicating that adult sera contain antibodies capable of recognizing both pre-F and post-F conformations (Figure 5C). We next compared the neutralizing titers against RSV A2-mKate measured by the fluorescence-based assay and the cell-ELISA platform. Although the cell-ELISA yielded modestly lower neutralizing titers than the fluorescence assay, it reliably discriminated differences in neutralizing capacity among individual sera (Figure 5D). These results collectively validate the platform's capacity to simultaneously evaluate conformational binding specificity and functional neutralization of RSV vaccine candidates.

## 4 Discussion

Pre-F-specific antibodies demonstrate superior neutralization capabilities against RSV (Magro et al., 2012; Mazur et al., 2023), underscoring the significant benefits of pre-F-based RSV vaccines, particularly for high-risk populations. Consequently, assessing the quality of serum antibodies elicited by RSV vaccine candidates is a key indicator of efficacy. Major parameters include the proportion of pre-F-specific antibodies and the breadth of neutralization against diverse RSV strains. Currently, soluble pre-F and post-F proteins engineered for structural stability (Marcandalli et al., 2019; Liang et al., 2024) are the standard for assessing post-vaccination serum antibody responses. Moreover, many studies employ engineered RSV strains to determine neutralization efficacy (Bakkers et al., 2024; Gidwani et al., 2024; Lin et al., 2025). However, despite the relative conservation of the F protein, viral escape mutants have been documented during the clinical application of monoclonal antibodies (Griffin et al., 2020). Therefore, developing methods to evaluate the neutralization potential of serum antibodies against wild-type clinical strains remains essential for RSV vaccine development.

Our previous studies demonstrated that strategic chemical treatments can stabilize the F protein in specific conformational states (Zhang et al., 2019; Lin et al., 2022). We have confirmed that HEp-2 cells infected with RSV express the F protein on their surfaces. Furthermore, treating these cells with varying concentrations of formaldehyde or methanol efficiently stabilizes the F protein in distinct conformations while preserving its antigenic epitopes. We employed the engineered RSV A2-mKate strain to develop a cell-ELISA platform, enabling visualization of RSV infection levels and determination of the appropriate multiplicity of infection (MOI). Although HEp-2 cells were infected with RSV A2-mKate at MOI of 2, flow cytometry detected only approximately 50% mKate-positive cells. The use of a higher infection dose did not yield a significantly increased proportion of positive cells, a finding consistent with the observed fluorescence intensity data. Importantly, the level of infection achieved was sufficient for all subsequent functional assays. For different wild-type RSV strains, variations in cellular infectivity necessitate precise MOI calibration to ensure the expression of saturating levels of F protein on the membrane surface of infected cells (Mclellan et al., 2013; Horie et al., 2016).

We evaluated various concentrations of formaldehyde and methanol for treating RSV-infected cells, using pre-F- and post-F-specific antibodies to assess the conformational states of the F protein on the cell surface. Our results indicate that a 4% formaldehyde treatment of infected cells favors the pre-F conformation of the F protein, although a proportion of post-F protein persists. Cells treated with 0.5% formaldehyde did not exhibit the post-F protein conformation, but the inadequate fixation on 96-well plates reduced the sensitivity for detecting pre-F specific antibodies. 60% methanol ensured complete presentation of the post-F protein conformation, effectively evaluating the binding titer of post-F protein-specific antibodies. Given that 4% formaldehyde-treated infected cells robustly display the F protein (both pre-F and post-F) and offer heightened detection sensitivity for cross-reactive antibodies (including 1129, MPE8, and 101F), we selected this condition for our cell-ELISA neutralization platform development. Notably, the MPE8 antibody exhibited pronounced reactivity with the F protein, and its use as the detection antibody in our neutralization assays enhances the sensitivity of the cell-ELISA platform. In our experiments, we directly compared the sensitivity of the cell-based ELISA neutralization platform using cells fixed with 4% formaldehyde vs. 60% methanol. The results consistently demonstrated that 4% formaldehyde fixation afforded superior sensitivity for detecting neutralizing antibody titers.

We conducted a parallel analysis of the neutralization titers for specific monoclonal antibodies and sera from healthy adult donors using both the cell-ELISA neutralization platform and a fluorescence-based neutralization assay (Sun et al., 2018). Despite lower neutralization potency observed for sera in our method, the approach effectively distinguished the differential neutralization capacities among various sera. To assess serum antibody quality post-immunization with pre-F and post-F proteins, we employed the cell-ELISA platform to evaluate the serum antibody response against the wild-type RSV (A2), successfully discerning the differences in serum antibodies elicited by different RSV vaccines.

This study acknowledges some limitations. Although other fixatives like paraformaldehyde and acetone have been used to fix surface F proteins (Krarup et al., 2015; Zhang et al., 2019; Zohar et al., 2022), we focused exclusively on formaldehyde and methanol and did not exhaustively explore methanol concentration gradients. Additionally, variations in treatment duration may impact the conformational state of the F protein. In our neutralization assay comparisons, we focused on F protein-specific antibody differences but did not extend the evaluation to a broader panel of post-vaccination sera. Although we assessed the quality of serum antibodies post-immunization with pre-F and post-F proteins using the cell-ELISA platform, comparisons across serum antibodies from different pre-F protein candidate vaccines have not been performed.

In summary, we have developed a cell-ELISA platform utilizing cell-surface F proteins to assess both the binding titers of antibodies specific to different F protein conformations and their ability to neutralize wild-type RSV strains. This methodology provides a rapid and effective tool for evaluating the antibody quality of RSV vaccine candidates, with particular utility for testing against diverse clinical strains.

## Data Availability

The original contributions presented in the study are included in the article/supplementary material, further inquiries can be directed to the corresponding authors.
